# Crossover-Use of Human Antibiotics in Livestock in Agricultural Communities: A Qualitative Cross-Country Comparison between Uganda, Tanzania and India

**DOI:** 10.3390/antibiotics11101342

**Published:** 2022-09-30

**Authors:** Jessica Myers, Mathew Hennessey, Jean-Christophe Arnold, Kayley D. McCubbin, Tiziana Lembo, Ana Mateus, Freddy Eric Kitutu, Indranil Samanta, Eleanor Hutchinson, Alicia Davis, Blandina T. Mmbaga, Fortunata Nasuwa, Meenakshi Gautham, Siân E. Clarke

**Affiliations:** 1 Department of Disease Control, London School of Hygiene & Tropical Medicine, Keppel Street, Bloomsbury, London WC1E 7HT, UK; 2 Veterinary Epidemiology, Economics and Public Health Group, Royal Veterinary College, Hawkshead Lane, Hatfield AL9 7TA, UK; 3 Department of Production Animal Health, Faculty of Veterinary Medicine, University of Calgary, 2500 University Drive NW, Calgary, AB T2N 1N4, Canada; 4 School of Biodiversity, One Health & Veterinary Medicine, University of Glasgow, Glasgow G12 8QQ, UK; 5 School of Public Health, Makerere University, New Mulago Hill Road, Kampala P.O. Box 7072, Uganda; 6 Department of Veterinary Microbiology, West Bengal University of Animal and Fishery Sciences, Kolkata 700037, India; 7 Department of Global Health and Development, London School of Hygiene & Tropical Medicine, Keppel Street, London WC1E 7HT, UK; 8 School of Social and Political Sciences, University of Glasgow, Glasgow G12 8RT, UK; 9 Kilimanjaro Clinical Research Institute, Moshi P.O. Box 2236, Tanzania; 10 Department of Paediatric and Child Health, Faculty of Medicine, Kilimanjaro Christian Medical University College, Moshi P.O. Box 2240, Tanzania

**Keywords:** antibiotic use, antibiotic resistance, crossover-use, antibiotic stewardship, India, Uganda, Tanzania, One Health, qualitative

## Abstract

Antibiotic use in animal agriculture contributes significantly to antibiotic use globally and is a key driver of the rising threat of antibiotic resistance. It is becoming increasingly important to better understand antibiotic use in livestock in low-and-middle income countries where antibiotic use is predicted to increase considerably as a consequence of the growing demand for animal-derived products. Antibiotic crossover-use refers to the practice of using antibiotic formulations licensed for humans in animals and vice versa. This practice has the potential to cause adverse drug reactions and contribute to the development and spread of antibiotic resistance between humans and animals. We performed secondary data analysis of in-depth interview and focus-group discussion transcripts from independent studies investigating antibiotic use in agricultural communities in Uganda, Tanzania and India to understand the practice of antibiotic crossover-use by medicine-providers and livestock-keepers in these settings. Thematic analysis was conducted to explore driving factors of reported antibiotic crossover-use in the three countries. Similarities were found between countries regarding both the accounts of antibiotic crossover-use and its drivers. In all three countries, chickens and goats were treated with human antibiotics, and among the total range of human antibiotics reported, amoxicillin, tetracycline and penicillin were stated as used in animals in all three countries. The key themes identified to be driving crossover-use were: (1) medicine-providers’ and livestock-keepers’ perceptions of the effectiveness and safety of antibiotics, (2) livestock-keepers’ sources of information, (3) differences in availability of human and veterinary services and antibiotics, (4) economic incentives and pressures. Antibiotic crossover-use occurs in low-intensity production agricultural settings in geographically distinct low-and-middle income countries, influenced by a similar set of interconnected contextual drivers. Improving accessibility and affordability of veterinary medicines to both livestock-keepers and medicine-providers is required alongside interventions to address understanding of the differences between human and animal antibiotics, and potential dangers of antibiotic crossover-use in order to reduce the practice. A One Health approach to studying antibiotic use is necessary to understand the implications of antibiotic accessibility and use in one sector upon antibiotic use in other sectors.

## 1. Introduction

Antibiotic resistance (ABR) is a major health challenge globally; it was estimated that 1.27 million human deaths were attributable to ABR in 2019, with sub-Saharan Africa and South Asia carrying the highest burden [1]. Antibiotic use (ABU) is a key driver of ABR development, exerting selective pressure on bacterial populations and contributing to survival and proliferation of bacteria with resistance traits [2]. Overuse of antibiotics, in humans, animals and plants, accelerates the development of ABR among bacterial populations, jeopardising antibiotic effectiveness and rendering infections untreatable [3,4]. Between 2000 and 2015, global antibiotic consumption in humans increased by 65% [5]. Although high-income countries (HICs) remain among the greatest consumers, this increase was primarily driven by low- and middle-income countries (LMICs) that face a high infectious disease burden and increasing household income [5,6,7]. The positive relationship between increasing income and antibiotic consumption rates found in LMICs is likely due to enhanced accessibility to goods and services, including medicines [8].

High ABU in the livestock sector is further reason for concern for ABR; global animal antibiotic consumption is predicted to increase by 67% between 2010 and 2030 [9]. This increase is expected to occur due to a higher demand for animal protein from a growing human population and subsequent intensification of livestock production [9]. As ABU increases, driving greater ABR development, LMICs experience a disproportionately greater ABR burden, whilst also facing inadequate access to quality healthcare, animal health services and antibiotics, particularly second line treatments for resistant infections [1]. Consequently, ensuring adequate access to antibiotics for humans and animals is vital for treating bacterial infections, but optimising use to reduce ABR risks is a major global health challenge [10]. Recent data from the World Organisation for Animal Health (WOAH) have shown a decrease in ABU in animals [11]. However, research and surveillance of ABU and ABR in animal production is largely focused on large scale commercial farms and rarely considers ABU in small scale backyard animal production settings that are significant for domestic consumption. Smallholder farms make up a substantial proportion of the agricultural community in LMICs [12]. Farms smaller than two hectares produce about 30% of food in sub-Saharan Africa, South East Asia and South Asia and are responsible for over 25% of livestock production in these regions [13]. ABU in these low-intensity production settings impacts a significant proportion of people and requires a greater understanding [13].

The challenge of ABR requires a collaborative One Health approach that acknowledges connections between human health, animal health and the environment, and seeks input and decision-making across sectors, ensuring any output considers the needs of all stakeholders [14]. The Global Action Plan (GAP) on Antimicrobial Resistance (AMR) identifies the One Health paradigm as a key strategy to mitigate ABR [15]. Following the GAP priorities, country-specific National Action Plans (NAPs) have been developed addressing AMR across human, animal and environmental sectors to improve antibiotic stewardship as well as the monitoring and surveillance of ABU and ABR. NAPs include the objective of optimising ABU in animals and humans, an objective that requires a nuanced understanding of local drivers of current ABU, particularly in contexts where the health of people and their livestock are closely connected, as is the case in LMICs [16,17,18].

Antibiotic crossover-use is defined as the practice of using antibiotic formulations licensed for humans in animals and vice versa [19]. To date, crossover-use of human antibiotic formulations in animals has been reported in agricultural communities in Bangladesh, Cambodia, Ethiopia, Guatemala, India, Nigeria and Uganda [19,20,21,22,23,24,25,26,27]. Although many of the same antibiotic classes are approved for use in humans and animals, antibiotic crossover-use could lead to adverse reactions in the animal due to excipients or potential overdose, and ABR development within the animal population due to subtherapeutic dosing [28]. Previous reports of crossover-use span a range of countries, but cross-country comparisons are not available to date and descriptions of antibiotic crossover-use drivers are scarce. To enhance our understanding of ABU at community level it is necessary to understand similarities and differences across different country contexts. This evidence will contribute to development of strategies for optimising ABU in both animal and human populations.

Therefore, the aim of this study was to provide a cross-country analysis of antibiotic crossover-use in rural, low-intensity production agricultural settings in LMICs, exploring characteristics and drivers which are common and unique across countries. The analysis specifically focused on the use of human antibiotic formulations in animals by livestock-keepers (LK) and medicine-providers (MP) in rural, agricultural districts in Uganda, Tanzania and India where there is limited access to public healthcare services for humans and animals.

## 2. Results

Crossover-use of human antibiotic formulations to treat animals was reported in all three countries by the full range of stakeholders interviewed (livestock-keepers, veterinary medicine-providers and human medicine-providers, community health workers, and other key informants). In Uganda, accounts were unprompted and often more in-depth, stemming from focus group discussions (FGD) surrounding the stocking and sale of antibiotics by human and veterinary drug shops. Whereas descriptions of human antibiotic crossover-use in India and Tanzania were mostly prompted by specific questions surrounding the use of human antibiotics in livestock. The use of veterinary antibiotic formulations in humans was also reported in Uganda and Tanzania. This was not explored further in this study, but reports are summarised in Table S1.

### 2.1. Characterising the Practice of Human Antibiotic Crossover-Use

#### 2.1.1. How Medicine-Providers and Livestock-Keepers Describe Crossover-Use

The way in which participants described crossover-use varied between countries, and between participant type. In India, medicine-providers who reported crossover-use include private veterinarians, public-private veterinary paraprofessionals, para-vets, drug shops specialised in selling either veterinary or human medicines, informal providers of human health, and homeopaths. Many of these providers talked about how they use human antibiotics in animals themselves, and how they prescribe these antibiotics for livestock treatment. Accounts of crossover-use were described by the full range of providers but were viewed as more problematic when veterinarians or key informants were describing para-vets engaging in the practice. When livestock-keepers in India were asked about the use of human antibiotics in animals, they reported seeking help from human medicine-providers when their animals were sick. They frequently described using human antibiotics that the veterinary or human medicine-providers had given or prescribed for use in their livestock. They also reported using medicines they had at home because ‘it would probably work’ (LK9, India).

In Uganda, medicine-providers included both human and veterinary drug shops. Drug shops discussed crossover-use through recounting how others use human antibiotics, using phrases such as ‘this is used’ and ‘they [livestock-keepers] would give’. Human drug shops reported that livestock-keepers would demand a certain drug and would occasionally report back to them regarding the success of the treatment. Often the drug shop would not know livestock-keepers intended to use these antibiotics to treat their livestock. The drug shops talked negatively about crossover-use, and as though it were something which often happens out of their control or knowledge. One human drug shop referred to crossover-use as ‘cross-cut’ and said that there is ‘misuse’ of human and veterinary medicines (MP7-Human drug shop, Uganda)

“*People use these drugs interchangeably, so we have a very big problem.*”

—MP5-Veterinary drug shop, Uganda

“*The role of these people [veterinary drug shops] is to health educate clients [on] the danger of using human medicine to treat animals… So, their role is to tell people …not to go to human drugs shops to buy drugs to treat animals.*”

—MP1-Human drug shop Uganda

In Tanzania, medicine-providers who reported crossover-use include human and veterinary drug shops, community health workers and a nurse. Drug shops described how livestock-keepers use human antibiotics in animals and recounted customers explaining which human antibiotics work for which disease in livestock. Similar to drug shops in Uganda, providers in Tanzania reported they often do not know the intended use of the antibiotics they sell. When asked if there are any human medicines used in livestock, both medicine-providers and livestock-keepers often listed different antibiotics and the clinical signs they were used for in which animal species. The same antibiotics were mentioned frequently by different participants.

“*Like that he will tell you ‘give me doxy, I am going to give to chickens’. Now I am not sure because when he takes things like that you don’t know if he is going to consume [himself] or treat his chicken.*”

—MP3-Human drug shop, Tanzania

In all three countries, human antibiotic crossover-use was reported as a common occurrence. Some medicine-providers stated they had someone come to them that day to take human antibiotics for their livestock. Others discussed how there are many kinds of human antibiotics which are used to treat animals. One para-vet in India stated that he provides human norfloxacin for around three to four cases of diarrhoea per day.

“*Interviewer: Are there cases where one will buy human medicine, like chloramphenicol to give to poultry?*

*Respondent 1: (Laughter) It’s there and so common.*”

—MP2-Veterinary drug shop, Uganda

Descriptions of antibiotic crossover-use from medicine-providers and livestock-keepers make it clear that some livestock-keepers know which antibiotics they use to treat their animals for specific conditions, either by name or by appearance of the antibiotic.

“*They [livestock-keeper] will say ‘Musawo [doctor], give me medicine for chicken’. You will ask them what that medicine looks like. He will even know the colour of the drug, saying ‘I want the white capsules to give to chicken or a goat’.*”

—MP7-Human drug shop, Uganda

Despite the reported commonality of human antibiotic crossover-use, some participants in all three countries raised concerns about possible safety risks relating to antibiotic crossover-use. In-depth interviews (IDI) with both medicine-providers and livestock-keepers revealed how doses of human antibiotics would be changed for animal use, suggesting beliefs that one only needed to alter the quantity of a human antibiotic to make it suitable for an animal. Medicine-providers indicated the possibility of overdosing was considered, but a reduced dose would make the human antibiotics safe for use in animals. A medicine-provider explained that drugs for humans work in animals, “*by decreasing the dose, it works*” (MP4-Informal human health provider, India), while another described livestock-keepers asking for doxycycline for their chickens saying they “*will ask you for a half dose*” (MP3-Human drug shop, Tanzania).

#### 2.1.2. Animals Treated and Human Antibiotics Used in Crossover-Use

Participants described a wide range of animals being treated with human antibiotics, which may in part reflect differences in the livestock systems and cultures across the settings studied (Table 1). However, crossover-use in chickens and goats was frequently reported, across all three countries.

In all countries, respondents named a range of antibiotics when asked which human medicines were used in animals; they also often came unprompted. Amoxicillin, tetracycline and penicillin were reported as used in all three countries. In Tanzania, amoxicillin was frequently mentioned to treat chickens. In Uganda, chloramphenicol was reported as frequently used in chickens. India had the widest variety of antibiotics mentioned to treat animals, most commonly metronidazole.

Descriptions of crossover-use were not limited to antibiotics. Participants also reported using pain killers, antimalarials and antiretrovirals (ARV), among others, to treat livestock (Tables S2–S4). In Tanzania, descriptions of human antibiotic crossover-use often came alongside descriptions of herbal medicines. The latter were used in humans and animals, indicating that both herbal and allopathic medicines are perceived as effective for both humans and animals.

“*Some people have HIV so they will share ARVs with pigs at home because they have the mentality that when the human takes ARVs they grow fat, so they also try it on pigs.*”

—MP6–Veterinary drug shop, Uganda

Respondents often described the clinical signs that were treated with human antibiotic formulations. Those most commonly mentioned were diarrhoea, cough, and wounds. A complete summary of human antibiotic formulations reported as used in different animal species for different clinical signs can be found in Tables S5–S7.

### 2.2. Factors Influencing Antibiotic Crossover-Use

Thematic analysis of crossover-use accounts generated four major themes representing key drivers of antibiotic crossover-use across the three countries: (1) medicine-providers’ and livestock-keepers’ perceptions of the effectiveness and safety of antibiotics, (2) livestock-keepers’ sources of information, (3) differences in availability of human and veterinary services and antibiotics, (4) economic incentives and pressures. In the following section we discuss the nuanced differences between countries where appropriate and highlight connections between themes.

#### 2.2.1. Medicine-Providers’ and Livestock-Keepers’ Perceptions of the Effectiveness and Safety of Antibiotics

Perceptions of human and animal antibiotics, and the efficacy, potency, and safety of using different formulations were commonly given as justification for crossover-use in all three countries.

##### Human and Animal Antibiotics Are the Same

Frequently, across all three countries, livestock-keepers and medicine-providers said antibiotic crossover-use occurs because people do not perceive, or are not aware of, any difference between antibiotics for humans and animals, stating “*most medicines are the same*” (LK5, India). A medicine-provider said “*I think it’s lack of awareness*” (MP3-Human drug shop, Uganda) to explain why people buy chloramphenicol from human drug shops for animal use.

“*We just think that if it works for humans, it might work in the cows for the same problem. That’s what we think.*”

—LK7, India

##### Human Antibiotics Are More Effective and Better Quality

A widespread perception across all three countries was that human antibiotics treat disease in animals more effectively than animal antibiotics. Livestock-keepers described their success treating animals using human antibiotics, explaining “*they don’t get well*” with veterinary drugs, but “*when you give them the other one [human antibiotic] they get up*” (LK2, Tanzania). When questioned on the use of human antibiotics in animals, medicine-providers answered, “*it actually works*” (MP17-Human drug shop, India) and “*it gets some better result*” (MP15-Veterinarian, India). Medicine-providers also recounted stories they heard from livestock-keepers regarding greater effectiveness of using human antibiotics to treat animals. For example, one medicine-provider from a human drug shop recounted a livestock-keeper asking for antibiotics and telling them “*antibiotics meant for chicken don’t work*” (MP1-Human drug shop, Uganda).

One medicine-provider from a veterinary drug shop explained they thought livestock-keepers were finding success using human antibiotics as treatment because the veterinary antibiotics are over-used and no longer effective. The medicine-provider described ABU on larger scale farms to treat and prevent disease, and the description alluded to an understanding of over-use causing reduced effectiveness of veterinary antibiotics.

“*Even people who rear chicken on large scale do this [use human antibiotics] but what brings this about is using antibiotics when chickens are not sick so when they use enrofloxacin for human they will be trying to look for an antibiotic that can cover the diseases they want to treat, because these [veterinary antibiotics] are over used and the [chicken’s] bodies are now used to them.*”

—MP6-Veterinary drug shop, Uganda

Participants in India linked the perception of human antibiotics being more effective with the view that human drugs are of better quality.

“*I have seen some human medicine works very much in animals. I have seen in mastitis my medicine is not working but [brand name redacted], amoxicillin and clavulanic acid (human antibiotic) works. Quality of human antibiotics is better.*”

—MP9-Para-vet, India

##### Safety Considerations

It was evident that safety concerns were shaping when and how participants practiced antibiotic crossover-use, as medicine-providers described it as an act of last resort if they deemed the health of the animal was seriously compromised.

*"A person came to me and asked, ‘Doctor my goat is having loose motion, what can we do?’ If I see the condition of the goat is really bad and it might die without a treatment, I may ask him to have a human medicine of a low dose.*”

—MP1-Informal human health provider, India

Despite reported regularity of antibiotic crossover-use, both human and animal medicine-providers commented on the danger of providing animals with human antibiotics. Drug shops in Uganda reported customers would hide the intended use of drugs they were buying, indicating a perception that it was wrong to use these in animals. One medicine-provider explained that “*the client will lie to you that the drugs are for humans, yet he/she is going to give the drugs to the birds*” (MP1-Human drug shop, Uganda).

It was clear in India that livestock-keepers and medicine-providers would balance the risks and benefits of antibiotic crossover-use as they reported that it was acceptable for mild clinical signs of disease, “*but if it is severe then we have to go to the veterinary doctors*” (LK3, India). A reported deterrent for use of human drugs in animals was the potential risk to a medicine-provider’s reputation if the human drugs did not work or harmed the animal.

“*We don’t want to take that risk. Maybe the cow was going to die anyway, but if it dies after taking medicines from my store it could create a problem. We still give medicine if there’s an emergency. Otherwise, it’s preferred that you have a prescription from a veterinarian…In case he isn’t there, we try and give medicines understanding the symptoms, we suggest the dose.*”

—MP17-Human drug shop, India

#### 2.2.2. Livestock-Keepers’ Sources of Information

Livestock-keepers’ participation in the practice of antibiotic crossover-use was reportedly influenced by multiple sources of information.

##### Trial and Error and Word of Mouth

Interviews with medicine-providers and livestock-keepers suggested that antibiotic crossover-use by livestock-keepers often occurs through an experimental process of trial and error. Respondents explained that when they were unable to get veterinary antibiotics, for availability or economic reasons, they would try human antibiotics. One medicine-provider of human health recounted a livestock-keeper saying “*[veterinary] doctors said they don’t have, so we came to you for the meds. And they worked.*” (MP4-Informal human health provider, India). A veterinary drug shop described this as a “*discovery*” which is continued when it is seen to be effective (MP2-Veterinary drug shop, Uganda).

Success of this trial-and-error approach to using human antibiotics in animals generated the positive perceptions of effectiveness, safety and quality reported in the previous themes. Participants consistently reported word of mouth as a driving force for others to partake in crossover-use, further cementing these perceptions. A livestock-keeper commented that their “*fellow member, mother and colleague*” talk about using human antibiotics in animals (LK4, Tanzania). Participants described that word of mouth has driven crossover-use for many years.

“*Even me when we were still young, we bought human antibiotics and gave it to chicken…The veterinary doctor came and immunized our chickens and they failed to respond, so the neighbours to mammy told her to buy CAF [chloramphenicol] and give it to them or else they will die. Mummy bought the drug, and we gave them, and they got healed. Since we never called the veterinary doctor again.*”

—MP4-Human drug shop, Uganda

Medicine-providers linked the sharing of “*success stories*” to people going directly to drug shops knowing what they want to buy for their animals without seeking advice from healthcare professionals, formal or informal (MP2-Veterinary drug shop, Uganda). Medicine-providers described these customers as having a “*biased mind*” as they insist on buying human drugs that they had been told are effective in treating their animals (MP5-Human drug shop, Uganda).

“*She will come and place an order for more drugs and tries to explain how the capsules look like, if you ask why she’s taking them, and then she will tell you ‘that I give to poultry, and they respond well to it’.*”

—MP3-Human drug shop, Uganda

##### Advice from Medicine-Providers

Indian livestock-keepers commonly reported that informal animal health providers (para-vets) were influential in their participation in crossover-use. One livestock-keeper explained that when they did not have time to go to the government veterinarian, they would contact an informal provider of veterinary antibiotics who would “*perhaps double the dose of human medication for the cows*” (LK10, India). Medicine-providers themselves, both formal and informal, reported giving or prescribing human antibiotics for use in animals.

“*I don’t know much about medicines. The veterinary doctor tells me that some medicines work the same for animals and humans. So, they give the medicines.*”

—LK5, India

#### 2.2.3. Differences in Availability of Human and Veterinary Services and Antibiotics

Several factors influencing the availability of both human and veterinary antibiotics to livestock-keepers and medicine-providers were described across all countries as reasons for use of human antibiotics in animals.

##### Veterinary Services Less Easily Accessible than Human

Study participants across all countries stated access to veterinary services was limited, particularly in comparison to human health services. Medicine-providers reported having to travel long distances to veterinary drug stockists. They described this as time-consuming so, instead, they bought from human drug shops which were closer to “*get the work done*” (MP15-Animal development volunteer, India). Livestock-keepers also reported being deterred from travelling long distances to the veterinarian because “*getting there… it will not be for free*” (LK1, Tanzania). Livestock-keepers explained that they go to see human health providers for their animals as the government veterinarian is not always available; stating the veterinarian would “*only come on specific days*” (LK9, India).

##### Lack of Desired Veterinary Antibiotic Formulations

Even when the veterinarians were available, they did not always have medicines to dispense, or were unwilling to open large cans for treating one or more small animals. They ended up prescribing antibiotics (sometimes human ones) that livestock-keepers then had to buy from drug shops. Medicine-providers in India reported that human antibiotics were administered to animals because veterinary formulated antibiotics were unavailable. One para-vet simply stated “*no veterinary formulation of [brand name redacted] norfloxacin-tinidazole is available*” when asked why they were using the human formulation in animals (MP8-Para-vet, India). A private veterinarian explained they thought para-vets used human antibiotics due to unavailability of veterinary antibiotics, stating “*it’s difficult to buy ceftriaxone of the veterinary company. So, if they [para-vet] see the human ceftriaxone, they will buy it*” (MP14-Private veterinarian, India). Formal medicine-providers reported it was likely that informal medicine-providers gave human antibiotics to animals because they were “*very easily available*” (MP14-Private veterinarian, India). A private veterinarian in India told that despite availability of veterinary formulated antibiotics improving in recent years as a result of an increase in manufacturing companies, some human drugs were still used due to unavailability of a veterinary alternative.

“*Suppose my oxytetracycline get finished I have to go [to] Sarisha then. But if there is human oxytetracycline nearby then I use that.*”

—MP12-Animal development volunteer, India

In India, public and private veterinarians also spoke of limited supplies of medicines from government sources, and how this led to providers resorting to the use of human formulations from private drug shops to fill the gap.

*"The government is giving medicines in a limited way. So that is how the human thing is coming to veterinary practice.*”

—MP14-Private veterinarian, India

##### Unsuitable Packaging Size

In other cases, veterinary formulations might be available, but participants reported the packaging size as unsuitable and/or uneconomic for use in animals of smallholder farmers. Medicine-providers reported that there are many small-scale farmers in the village, but the “*veterinary drugs have no small packages*” (MP2-Veterinary drug shop, Uganda). They explained this drives antibiotic crossover-use because smaller quantities can be obtained from human drug shops at a lower price.

One medicine-provider from a veterinary drug shop commented on the economic implications of small packaging sizes, explaining that they must recover the money for the whole opened package despite only using a portion of the contents. This negatively impacts livestock-keeper’s ability to access the veterinary medication they need at an affordable price.

“*Another challenge is that when we go to treat animals, once you open an ampoule of a given drug it must be used within 7 days before it goes bad or it gets spilt, yet we do not use it all at once, so it usually goes bad, and we tend to exploit the farmers in terms of cost recovery in the business aspect.*”

—MP2-Veterinary drug shop, Uganda

##### Human Antibiotics Easy to Acquire over the Counter

Some participants reported acquiring human antibiotics over the counter (OTC) was comparatively easier than accessing veterinary antibiotics. Medicine-providers commented that the lack of prescription required to sell human antibiotics allowed customers to self-medicate and facilitated the practice of crossover-use.

“*You don’t need a prescription to dispense… there are many shops, human chemist shops, that deal with [human] antibiotics, and they will take it out and give it to the para-vets. It is very easily available, it’s easily available.*”

—MP14-Private veterinarian, India

Veterinary medicine-providers suggested that if prescriptions for human antibiotics were required by all medicine-providers, then competition amongst providers might be minimised, reducing pressure on medicine-providers to sell human antibiotics for animal use.

“*For me, what I think would be our role as both human and veterinary system is to guide our clients before we sell the drugs to them. More so the human drugs would only be sold on prescription only, but this open system of ours is the one causing problems, where people come in as they wish, buy drugs, and take.*”

—MP2-Veterinary drug shop, Uganda

#### 2.2.4. Economic Incentives and Pressures

Participants reported economic factors as influencing antibiotic crossover-use across all three countries.

##### Human Antibiotics Are Cheaper

For livestock-keepers, the higher cost of veterinary antibiotic formulations was a major factor contributing to the use of human antibiotics for livestock, and human drugs were consistently reported to be cheaper in all three countries. A medicine-provider said that sometimes people have no money to pay veterinary professionals, and therefore they use human medicines instead “*because human medicine is cheap*” (MP1-Veterinary drug shop, Tanzania). The lower cost was also often linked to the availability of smaller pack sizes of human antibiotics. Livestock-keepers commented that it was illogical to use animal medicine when the human medicine was much cheaper.

“*The human medicine costs lesser than animals, like the medicine for loose motion for human costs 2 rupees while tablet for cow one tablet costs 40–80 rupees. Why would we use that?*”

—LK1, India

##### Economic Incentives and Pressures for Selling Human Antibiotics for Animals

Economic incentives to sell human antibiotics for animal use were reported by medicine-providers in Uganda and India. In India, informal animal health providers reported selling antibiotics to increase their income, in addition to their primary role of vaccination and artificial insemination.

As previously described, providers of human antibiotics in Uganda reported that customers frequently demanded antibiotics to treat their livestock. Drug shop vendors explained that if they did not provide these antibiotics, they risked losing business. Thus, meeting patient demands was an economic necessity for them. One medicine-provider from a human drug shop recalled people asking for chloramphenicol to treat chickens and explained that “*if you refuse the customer will never come back*” (MP1-Human drug shop, Uganda). Another human medicine-provider explained the perceived necessity of giving customers the requested human antibiotics for animal use, despite reservations, to ensure their customers did not go to their competition to source the antibiotics instead.

“*They will say, ‘Give me chloramphenicol. Give me amoxicillin’… ‘I am going to give it to chicken.’ And you will tell them that it is not for chicken. And they will say, ‘Just give me. Me I want to use it.’ Because we are in a competitive society and people have to look for bread, you end up giving out. Because if you don’t, what will you eat? What will you use to pay the bills?*”

—MP7-Human drug shop, Uganda

Reports of livestock-keepers perceiving human antibiotics as more effective, better quality, and cheaper allow us to understand why there is this demand from customers for human antibiotics. For each of the themes discussed above, additional illustrative quotes are shown in Table 2.

#### 2.2.5. Summary of Factors Influencing Crossover-Use

The breadth of the themes identified shows multiple factors play a role in influencing human antibiotic crossover-use in animals, with commonalities identified in the three countries (Table 3).

**Figure 1:**
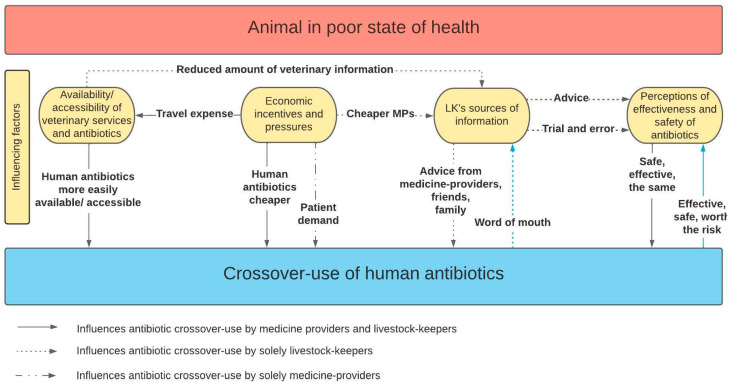
Schematic showing the factors influencing medicine-providers and livestock-keepers in partaking in the practice of crossover-use.

The four identified themes are distinct yet interconnected in their influence on antibiotic crossover-use, as displayed in Figure 1. For example, livestock-keepers’ economic considerations affect access to veterinary services, through the ability to pay for travel costs and the level of veterinary service people could afford. Consequently, access and availability of veterinary services affect sources of information; if a veterinarian could not be accessed or was not available, people may resort to information provided by friends and family. The sources of information people access shape perceptions around human and animal antibiotics and antibiotic crossover-use and may drive the practice. Medicine providers’ economic considerations influence what they sell or prescribe to livestock-keepers due to patient demand, or travel expenses to acquire veterinary antibiotics. Similarly, availability and accessibility of veterinary antibiotics affects what medicine-providers sell or prescribe to livestock-keepers.

##### Human Antibiotics Used in Crossover-Use

A total of 26 different human antibiotics were described in the transcripts as being used in animals, though the antibiotic types varied between countries (Table 4). In Uganda, chloramphenicol was the most reported antibiotic, mentioned in all FGD transcripts. In India, the most commonly mentioned antibiotic was metronidazole (17%) whilst in Tanzania, it was amoxicillin (59%). Use of human amoxicillin was reported in all three countries, mentioned in about a third (31%) of transcripts overall, making it the most commonly reported antibiotic.

Nine of the 26 human antibiotics found to be used in animals in this study are highest priority critically important antibiotics (CIAs) for human health in the fluoroquinolone, macrolide and third generation or higher cephalosporin classes of antibiotics, or combinations of. A further five are high priority CIAs for human health in the penicillin and aminoglycoside classes of antibiotic. In addition, eight of the total antibiotics are ‘Watch’ category antibiotics in WHO AwaRe list, and two combinations categorised as ‘not recommended’ [29]. Chloramphenicol is also not recommended for use in animals due to potential development of aplastic (non-regenerative) anaemia upon human consumption of animal products containing traces of the antibiotic.

## 3. Discussion

This study aimed to investigate the practice of crossover-use of human formulation antibiotics in animals in low-income, low-intensity production agricultural communities in Uganda, Tanzania and India, and to understand contextual drivers of this practice. We demonstrate how antibiotic crossover-use of human formulations in animals occurs in all study settings and, that this practice was influenced by a similar set of parameters across the three study sites, though some contextual differences also exist. Participants described antibiotic crossover-use mostly in poultry (Uganda and Tanzania) and goats (India) and to a lesser extent in other species. A wide range of antibiotics were mentioned including nine highest priority CIAs, seven of which were reported solely in India. According to our study participants, chloramphenicol was the human antibiotic most commonly used in animals in Uganda, and metronidazole in India. Amoxicillin was the most reported human antibiotic used in animals in Tanzania and overall, across the countries. The study participants frequently reported diarrhoea, cough and wounds as treated with human antibiotics. The fundamental pattern observed across study sites is that crossover-use occurs due to multiple interconnected contextual drivers, demonstrated in the generated themes: (1) medicine-providers’ and livestock-keepers’ perceptions of the effectiveness and safety of antibiotics, (2) livestock-keepers’ sources of information, (3) differences in availability of human and veterinary services and antibiotics, (4) economic incentives and pressures. The occurrence of antibiotic crossover-use as noted in these three countries should be taken into consideration and further explored when developing interventions to promote antibiotic stewardship in rural, low-intensity production agricultural communities in LMICs.

The design of this study as a secondary data analysis of datasets which examined antibiotic availability and use in agricultural communities more broadly, presented a limitation in that antibiotic crossover-use was often only briefly mentioned or not fully explored, reducing the depth of insight into the practice. However, this is the first cross-country comparison characterising crossover-use and the factors driving it. The accounts of antibiotic crossover-use cannot be used to measure the full extent or frequency of the practice. The variation in the amount of data from each country, and the qualitative nature of the design and objectives of the original studies means that quantification of the practice is not possible and further research is needed to do this.

The use of human chloramphenicol in animals, reported in this study in both Uganda and India, has also been previously documented in commercial poultry farming systems in Cambodia, and Nigeria meaning that antibiotic crossover-use may not be a phenomenon unique to small-scale backyard farming. In Nigeria it was found that human chloramphenicol was more likely to leave residues in poultry eggs intended for human consumption than veterinary formulations of chloramphenicol [20,21]. Toxicity to humans from chloramphenicol residues in animal products is a concern due to the risk of aplastic (non-regenerative) anaemia developing after consumption of its residues in food products of animal origin, and its potential carcinogenicity; in many countries, use of chloramphenicol in livestock is banned as a consequence [19,30,31]. Omeiza et al. (2012) found that only 26.7% of the Nigerian farmers involved in their study were aware that chloramphenicol is discouraged for use in food producing animals [20]. The most commonly mentioned human antibiotic used in animals across all three countries in this study was amoxicillin, a high priority CIA. Use of human formulation amoxicillin has also been reported in poultry in Cambodia and Guatemala [21,23]. Amoxicillin and other CIAs identified among the human formulation antibiotics used in animals in this study is concerning considering the potential for development and spread of resistant microbes within the animal population through the food chain and via the environment through animal waste [32,33,34]. Inaccurate dosing is a major concern of crossover-use, with risk of toxicity and ABR development. Considerations of overdose and dosing differences, when practicing crossover-use, were evident across the three countries, indicating some level of awareness of different formulations, though this may not reflect understanding of the pharmacokinetics of the drugs. Training initiatives to improve understanding of the risks and highlight unknown effects of crossover-use would be beneficial to raise awareness among medicine-providers and livestock-keepers about potential harms and reduce this practice.

We identified understanding of and perceptions of differences between human and animal antibiotics as a driver of antibiotic crossover-use in this study. The belief that if an antibiotic works in humans it will work in animals was previously described in India by Arnold et al. (2021) [26]. Our study shows this understanding to be a common driver also in Tanzania and Uganda. Snively-Martinez (2019) described that poultry smallholders in Guatemala believe poultry and human antibiotics are different but work in similar ways, and that human drugs are just as effective in treating poultry diseases [23]. The belief of greater effectiveness of human antibiotics for treating livestock was reported in all three countries in our study and was described as having been established through trial and error. In a study of ABU in Cambodia, commercial farmers similarly stated that use of human antibiotics was based on their own past experiences [21]. These perceptions of greater effectiveness of human antibiotics require further study to understand how the antibiotics are being used. A further area of investigation is whether local data on ABR in animal populations is consistent with this perception that veterinary antibiotics are less effective, and the implications of this for future treatment of livestock.

The drivers of antibiotic crossover-use identified in this study are consistent with the literature on self-medication in a range of agricultural settings of Uganda, Tanzania, India and Bangladesh. These include: limited access to healthcare facilities, customer demand, habit and drug preference, financial benefit for customers and dispenser, and limited awareness of safety concerns around self-medication [35,36,37,38,39]. It appears the additional influencing factors unique to crossover-use are the comparatively reduced availability and the unaffordability of veterinary drugs and the perceptions surrounding the differences between human and veterinary drugs. This finding is consistent with those in Guatemala, where ethnographic decision modelling showed that these issues drove the use of human instead of veterinary antibiotics in poultry among smallholders [23]. There, travel distance and associated time and income loss were identified as key to the decision to use human antibiotics. Similarly, a study investigating ABU in different agro-ecological production systems in Ethiopia, reported crossover-use in sites where access to veterinary drugs and government veterinarians was limited [25]. Another recent study in Uganda found that human antibiotics were given to animals because human drug shops were closer to people’s homes, and because of a widespread perception that human antibiotics are effective in animals [27]. A continuous narrative in the present study is the ability to purchase antibiotics OTC without prescription enabling participants to buy what they wish, irrespective of intended use. This is a wide-spread practice and a well-recognised stewardship challenge for policymakers in the countries studied [40,41,42]. The economic incentive to sell human antibiotics for use in animals, despite provider reservations, was influenced by the competitive nature of the private sector. Similarly, Dione et al. (2021) found this was a strong determinant of inappropriate retail sales in their veterinary drug supply chain analysis [43]. Our findings indicate that in order for existing regulations on prescriptions to be a deterrent for crossover-use, there needs to be training of veterinary and human healthcare providers who are able to act as antibiotic gatekeepers. However, it appears that fundamental structural change to improve access and affordability of veterinary formulations suitable for use by smallholder producers will also be needed in order to reduce the practice of crossover-use among this population. In the short term, it would be beneficial to focus on the development of interventions that will discourage the use of highest and high priority CIAs for human health, given the potential negative public health implications with respect to the development and spread of ABR to these vital drugs.

Through adopting a One Health approach, antibiotic crossover-use was identified in all the rural, agricultural communities included in this study. Thus, by simultaneously investigating ABU in both animal and human sectors, antibiotic crossover-use may be identified in other countries where it may have been previously overlooked when assessing ABU in each sector separately. Veterinary and human antibiotics are prescription only medication in all three countries, with the exception of antibiotics ‘when contained in preparations for animal feeding stuffs’ in Uganda [44,45,46,47]. Yet, there is evidently a disconnect between regulation and practice. This disconnect highlights the importance of utilising a One Health approach at all stages of policy development on ABU: the design of investigations into ABU, surveillance of ABU and awareness raising on responsible and prudent ABU, as well as development, packaging and sales of antibiotics by pharmaceutical manufacturers and healthcare providers. Policies surrounding ABU developed in one sector may impact upon ABU in other sectors. For example, in Tanzania, human accredited drug dispensing outlets are allowed to dispense selected antibiotics, whereas veterinary accredited drug dispensing outlets are no longer able to do so [45]. Therefore, people resort to using human doxycycline and amoxicillin in animals as these are still available in human drug outlets [48]. Furthermore, veterinary drug shops in Uganda are not permitted by the National Drug Authority to sell veterinary formulation chloramphenicol due to its human health risks, yet our findings show that chloramphenicol continues to be used in animals in human formulation [19]. This highlights that restricting access of certain drugs in one sector will impact on use in the other sector and vice versa.

This study provides evidence that there is a poor understanding of how antibiotics are used at the animal level in many LMICs, highlighting the need to survey and characterise modalities of ABU at the level of the end users, including crossover-use. Currently, ABU estimates in animal production systems are based solely on antibiotic sales data within the veterinary supply chain [49]. Surveillance of ABU requires a One Health approach; surveying human and animal ABU separately does not build a comprehensive picture of ABU. National Action Plans for AMR in Uganda, Tanzania and India all identify surveillance of ABU in human health and animal production as a key objective. A more integrated approach encompassing crossover-use is particularly important in these countries and in other LMIC contexts where health choices relevant to humans and animals are often interconnected. Future work into the practice of crossover-use could include farm and household surveys to quantify use at a granular level. This type of data would help inform the magnitude of the problem, and if combined with ABR data, help to build a more holistic picture of transmission dynamic pathways. However, the collection of this type of data is known to be difficult in resource scarce settings, such as India [50]. With inclusion of crossover-use data, countries will be able to build an accurate picture of ABU and ABR and develop more targeted interventions and awareness and education campaigns for antibiotic users for effective tackling of ABR in LMICs.

It would be tempting to conclude that lack of enforcement of regulation surrounding antibiotic sales allows crossover-use under the pressure of economic and availability factors in these settings. However, strengthening legislation without improving availability and affordability of the much-needed antibiotics in the animal health sector would not have the desired effect. Imposing restrictions and bans on the use of certain antibiotics, as is done in high income countries, is not feasible in LMICs as it is likely to exacerbate the inaccessibility of veterinary antibiotics, negatively impacting animal health and welfare due to restricted access to lifesaving medication, whilst also jeopardising food security and livelihoods. A challenge in the control of ABR in LMICs is regulating ABU while ensuring access to necessary treatment [51]. If access improves, the implementation of country-specific regulation, accounting for types of production systems, human and animal healthcare service capacities, socioeconomic context, and existing legal frameworks, will have a greater chance of success.

## 4. Materials and Methods

### 4.1. Study Design

This paper draws on qualitative cross-sectional data obtained from independent studies conducted in Uganda, Tanzania and India between 2017 and 2021. The scope and aims of these projects were wider than solely investigating crossover-use, but ABU in humans and animals and antibiotic stewardship were core topics of study in all four projects.

### 4.2. Study Setting

#### 4.2.1. Study Sites

We focused on rural, agricultural communities practicing livestock farming in Uganda, Tanzania and India. The Uganda site was the Luwero district, a rural agricultural district 62 km north of Kampala, the commercial and administrative capital of Uganda, with a combination of subsistence and small and large-scale commercial farming. The Indian sites were villages within two different Gram Panchayats (an administrative unit consisting of a cluster of villages) in the South 24 Parganas district of the state of West Bengal. Eighty-four percent of the population of South 24 Parganas live in rural areas where most of the population are marginal farmers, with only a subsistence income level [52]. In Tanzania, study sites included six villages in northern Tanzania, two each from the districts of Mwanga, Ngorongoro and Misungwi, within the Kilimanjaro, Arusha and Mwanza regions, respectively, representing the key livelihood strategies predominant in rural East Africa: rural smallholder, pastoral, and agro-pastoral.

The study sites are characterised by limited access to public healthcare services for humans and animals resulting in a higher reliance on the private healthcare sector for both [38,53,54]. For the majority of people living in rural communities in these three countries, private and informal providers are the first source of human and animal healthcare, including antibiotics [53,55,56,57]. In Uganda, at the time of data collection, 97 human drug shops and 21 veterinary drug shops were operating in the Luwero area. The two study sites in India had limited access to formal public or private veterinary services with the nearest formal private veterinarians between 3 km and 13 km away. There were multiple public-private and private veterinary paraprofessionals. Veterinary drug shops were between 3 km and 13 km away; access to human healthcare services was much greater; there were multiple human drug shops with a limited number also stocking veterinary medicines.

All three countries have reported increasing ABR, and increasing antibiotic consumption trends, particularly in animal agriculture [17,18,58,59,60]. Antibiotics are prescription only medications for humans and animals in the three countries, with the exception of veterinary antibiotics ‘when contained in preparations for animal feeding stuffs’ in Uganda, which are allowed to be sold OTC [44,45,46,47]. However, in all three countries, veterinary and human antibiotics are frequently acquired OTC [38,40,61,62].

#### 4.2.2. Study Respondents

We have used the term ‘medicine-providers’ to refer to a diverse range of providers that delivered healthcare and antibiotics to rural agricultural communities in the three countries. These included private and informal healthcare providers in the human and animal sectors, public-private veterinary paraprofessionals, and private professional veterinarians in India, human drug shops and veterinary drug shops in Uganda and Tanzania, and community health workers and nurses in Tanzania (Table 5).

We have used the term ‘livestock-keepers’ to define farmers who raise livestock mainly for their own consumption in India and community members in livestock keeping communities in Tanzania, where interviews explored healthcare for livestock and family members.

The term ‘key informants’ refers to other members of the study sites in India who were anticipated to have a high level of knowledge of the study communities, including details of livestock systems, related health practices, and antibiotic knowledge.

### 4.3. Data Collection

This study is a secondary analysis of IDI and FGD transcripts available through four independent but similar research projects in Uganda (*n* = 2), Tanzania (*n* = 1), and India (*n* = 1) conducted between 2017 and 2021. These studies all focused on drivers of antibiotic use in rural agricultural communities, and across humans and animals. IDIs and FGDs were conducted with human and animal medicine-providers, community members, livestock-keepers, and key informants. Topic guides were used in all countries and administered by researchers trained in qualitative techniques. Each topic guide was context specific. In Uganda, reports of antibiotic crossover-use were unprompted and came mainly after FGD participants were asked whether drug shops sold both human and animal antibiotics, and how antibiotics were being used in the community. From this, accounts of antibiotic crossover-use arose, and questioning of the diseases treated in which animals followed. In contrast, data collection in India focused specifically on antibiotic crossover-use, asking questions such as ‘do you stock any antibiotics for humans/animals’. When the practice was identified, participants were asked specifically about the species of animals for which they would provide human antibiotics and reasons for these. In Tanzania participants were asked whether there were any human drugs used to treat livestock disease and vice versa, about the animal species treated and the diseases treated.

Of the 108 transcripts received, 59 contained information relating to antibiotic crossover-use of human formulated antibiotics in animals and were eligible for inclusion in this study. Transcripts included mainly FGDs in Uganda, IDIs in India, and an equal mix of IDIs and FGDs in Tanzania (Table 6). Detailed methods from each study can be found in Supplementary Materials (Table S8).

### 4.4. Data Analysis

Reflexive thematic analysis was performed to generate themes demonstrating the drivers of antibiotic crossover-use [66]. We followed an experiential approach, taking what participants said they think, feel, and do as reality and conducted the analysis following the phases identified by Braun and Clarke (2006) [66,67,68]. First, data familiarisation was achieved by reading eligible transcripts; discussions amongst project teams aided in understanding the roles of different medicine-providers within each country context. An inductive orientation was taken to coding, using QRS International NVivo 12 (2020) for data management [69]. Similar codes were brought together to generate themes, and these themes were discussed with the research team and transcripts re-read to ensure the themes were representative of the data. We sought to identify common themes across the countries as well as themes unique to individual countries to understand human antibiotic crossover-use practices and their drivers in-depth. Finally, themes were organised to address the aim of the paper in the most cohesive and coherent manner possible.

## 5. Conclusions

This study provides evidence that antibiotic crossover-use of human formulations in animals occurs in rural, low-intensity production agricultural communities in Uganda, Tanzania and India. Study results indicated several animal species are treated with a range of different human antibiotic formulations. We demonstrate commonalities between the three countries regarding species treated and the human formulation antibiotics used. This study is the first to provide a cross-country comparison of contextual drivers of antibiotic crossover-use. These comparable drivers are demonstrated in the generated themes: (1) medicine-providers’ and livestock-keepers’ perceptions of the effectiveness and safety of antibiotics, (2) livestock-keepers’ sources of information, (3) differences in availability of human and veterinary services and antibiotics, (4) economic incentives and pressures. We argue that fundamental structural change to improve access and affordability of veterinary formulations suitable for use by smallholder producers is needed in order to reduce the practice of crossover-use. Therefore, further research with an emphasis on the availability and economic considerations of the use of human formulation antibiotics in animals is necessary to generate a more detailed understanding of the contextual drivers of crossover-use. Our findings also underscore the need for an integrated One Health approach in research, to investigate and understand ABU in both humans and animals in the same setting, in order to inform interventions to optimise antibiotic stewardship.

## Figures and Tables

**Table 1:** Animal species treated with human antibiotics in each country.

Species of Animal	Country		
	Uganda	Tanzania	India
Chicken	✓	✓	✓
Goat	✓	✓	✓
Turkey	✓	-	-
Duck	✓	-	-
Cow	-	-	✓
Dog	-	-	✓
Cat	-	-	✓
Sheep	-	-	✓
Not-specified	-	-	✓

**Table 2:** Additional illustrative quotes for common themes by country.

Theme	Sub-Theme	Quotes		
		Uganda	Tanzania	India
*Medicine-providers’ and livestock-keepers’ perceptions of the effectiveness and safety of antibiotics*	*Human and animal antibiotics are the same*	“People do not differentiate between drug shops, for one can come to a human drug shop and ask for combitic [penicillin & streptomycin], or for poultry drugs.”—MP4-Human drug shop	“You find one using medicines, which he has bought from the human being’s pharmacy shop. Because he just believes in antibiotic, then even human being antibiotic he goes to give to livestock.” –MP1-Veterinary drug shop	“Most of the medicines are same. Our medicines and cow’s medicines and dog’s medicines are the same. If they have fever, and we have fever, the medicine for the fever is the same.”—LK5
	*Human antibiotics are more effective*	“They [customers] will tell you they are going to give them to the chicken. They will tell you that antibiotics meant for chicken don’t work…human antibiotics tend to work on chicken more effectively.”— MP1-Human drug shop	“Chicken may be sick, cold/influenza… and these medicines from the veterinary officer…they do not get well. You use the one called doxy [doxycycline] which are human medicines, and the chicken get well.”—LK2	Interviewer: “Why Norfloxacin [human formulation] is used?” Respondent: “Norfloxacin gets some better result in case of goat.”—MP15-Veterinarian
	*Safety considerations*	“At times they give animals overdose because they don’t know the weight…so they don’t give them medicines basing on their weight, so they end up giving them overdose when they give them human drugs.”—MP5-Human drug shop	“He [customer] wanted two tablets of doxy… he says he is going to give to chickens… he will ask you for a half dose.” —MP3-Human drug shop	“A person came to me and asked, ‘Doctor my goat is having loose motion, what can we do?’ If I see the condition of the goat is really bad and it might die without a treatment, I may ask him to have a human medicine of a low dose… as I am not aware of the treatments, so I just give a low one.” —MP1-Informal human health provider
*Livestock-keepers’ sources of information*	*Trial and error (personal experience)*	“They [customers] do it as a discovery, they try it and see it working then they come and tell you their success stories that ‘Doctor for me I gave chloramphenicol to poultry, and it got better’.”—MP2-Veterinary drug shop	Not mentioned	“Sometimes when they don’t get meds then approach me. [Veterinary] Doctors said they don’t have so we came to you for the meds. And they worked.”—MP4-Informal human health provider
	*Word of mouth (Other livestock-keeper’s experience)*	Interviewer: “Now which diseases do farmers normally treat using the human drugs?” Respondent: “It should be fowl typhoid, because they usually develop diarrhoea with a white colour of their droppings and therefore if A used chloramphenicol and it worked for his poultry, he tells B and at times they don’t even know the name, but they say that ‘I gave white capsules’.”—MP2-Veterinary drug shop	Interviewer: “Right what other human medicine we use to treat animals?” Respondent: “All I know is that my fellow member, mother and my colleague talked about it.”—LK4	Not mentioned
*Differences in availability of human and veterinary services and antibiotics*	*Veterinary services less easily accessible*	“There are few veterinary drug shops compared to human drug shops.”—MP3-Human drug shop	“Livestock is near, a veterinary doctor is very far, if I find someone who can work on the problem that my livestock has, I will ask him to help.”—LK1	“If I find the antibiotic is not available in veterinary, then I use the human one.”—MP10-Veterinarian, India
*Economic incentives and pressures*	*Human antibiotics are cheaper*	“They say ‘Doctor your packaging is big and expensive, I will use 200 to buy [human] chloramphenicol capsule yet for you, you will sell to me 5,000 to 8,000’…that’s why they basically do it.”—MP2-Veterinary drug shop	“Some time you find he has no money to pay the expert when he comes to see his livestock…. That is why you find most of them using their own medicines… Because the human being’s medicines are cheap.”—MP1-Veterinary drug shop	“The human medicine costs lesser than animals like the medicine for loose motion for human costs 2 rupees while tablet for cow one tablet costs 40–80 rupees. Why would we use that?”—LK1
	*Economic incentives for selling human antibiotics for animals*	“Some people go and buy human antibiotics and they treat animals for example Caf [chloramphenicol] people give it to chicken…—And if you refuse the customer will never come back.”—MP1-Human drug shop	Not mentioned	“We are only supposed to do vaccination, and artificial insemination. But if we did just that we won’t make enough money, so we, on our own, have learnt how to use antibiotics from other veterinary doctors.“—MP16-Veterinary paraprofessional (pranibandhu)

**Table 3:** A comparison of the themes identified as driving or deterring crossover-use of antibiotics across countries.

Theme	Sub-Theme	Driver or Barrier to Antibiotic Crossover-Use	Reported in The Data		
			Uganda	Tanzania	India
Medicine-providers’ and livestock-keepers’ perceptions of the effectiveness and safety of antibiotics	Human and animal antibiotics are the same	Driver	✓	✓	✓
	Human antibiotics are more effective	Driver	✓	✓	✓
	Human antibiotics are better quality	Driver	-	-	✓
	Safety considerations		✓	✓	✓
	Human antibiotics are safe in animals	Driver	✓	✓	✓
	Human antibiotics are dangerous in animals	Barrier	✓	✓	✓
Livestock-keepers’ sources of information	Trial and error (personal experience)	Driver	✓	-	✓
	Word of mouth (other livestock-keeper’s experience)	Driver	✓	✓	-
	Advice from medicine-providers (‘expert’ opinion)	Driver	-	-	✓
Differences in availability of human and veterinary services and antibiotics	Veterinary services less easily accessible	Driver	✓	✓	✓
	Veterinary antibiotics less easily available	Driver	-	-	✓
	Unsuitable packaging size	Driver	✓	-	-
Economic incentives and pressures	Human antibiotics are cheaper	Driver	✓	✓	✓
	Economic incentives for selling human antibiotics for animals	Driver	✓	-	✓

**Table 4:** Human antibiotic formulations per class and agent reported as used in animals by country and their importance to human health.

		Country				
		Uganda (N = 7)	India (N = 29)	Tanzania (N = 22)	Total (N = 58)	
**Number of Different Antibiotics Mentioned**		8	25	8	26	
**Average number of different antibiotics mentioned per transcript (range)**		2.86 (1–4)	1.66 (1–5)	1.5 (1–3)	1.74 (1–5)	
**Antibiotic class**	**Antibiotic**	**Number of transcripts each antibiotic is mentioned in per country**				**WHO list of critically important AM**
		**Uganda** **n (%)**	**India** **n (%)**	**Tanzania** **n (%)**	**Total** **n (%)**	
**Fluoroquinolones**	*Ciprofloxacin* ^w^	1 (10)	1 (3)		2 (3)	Highest priority CIA
	*Norfloxacin* ^w^		2 (7)		2 (3)	Highest priority CIA
	*Enrofloxacin*	1 (10)	1 (3)		2 (3)	Highest priority CIA
	*Ofloxacin* ^w^		1 (3)		1 (2)	Highest priority CIA
**Macrolides**	*Azithromycin* ^w^		1 (3)		1 (2)	Highest priority CIA
	*Erythromycin* ^w^			4 (18)	2 (3)	Highest priority CIA
**Cephalosporins (3rd, 4th and 5th generation)**	*Ceftriaxone* ^w^		2 (6)		2 (3)	Highest priority CIA
	*Cefotaxime* ^w^		1 (3)		1 (2)	
**Aminoglycosides**	*Gentamicin* ^A^		2 (7)		2 (3)	High priority CIA
	*Amikacin* ^A^		1 (3)		1 (2)	High priority CIA
**Penicillin**	*Amoxicillin* ^A^	3 (43)	4 (14)	13 (59)	18 (31)	High priority CIA
	*Penicillin* ^A^	1 (10)	1 (3)	1 (5)	3 (5)	
	*Amoxicillin-Clavulanic Acid* ^A^		1 (3)		1 (2)	High priority CIA
	*Ampicillin* ^A^		2 (7)	2 (9)	4 (7)	High priority CIA
**Cephalosporins (1st and 2nd generation)**	*Cephalexin* ^A^		3 (10)		3 (5)	HIA
**Chloramphenicol**	*Chloramphenicol* ^1^	7 (100)	1 (3)		8 (14)	HIA
**Sulphonamides**	*Trimethoprim-sulfamethoxazole* ^A^	1 (10)	2 (7)		3 (5)	HIA
	*Sulfadimidine* ^A^		2 (7)		2 (3)	HIA
**Tetracyclines**	*Tetracycline* ^A^	5 (71)	2 (7)	4 (18)	11 (19)	HIA
	*Oxytetracycline* ^w^		2 (7)	2 (9)	4 (7)	
	*Doxycycline* ^A^		2 (7)	11 (50)	12 (20)	HIA
**Nitroimidazole**	*Metronidazole* ^A^		5 (17)	1 (5)	6 (10)	
	*Ornidazole* ^A^		1 (3)		1 (2)	
**Combination**	*Ofloxacin-ornidazole* ^NR^		3 (10)		3 (5)	Highest priority CIA
	*Norfloxacin-Tinidazole*		3 (10)		3 (5)	Highest priority CIA
	*Ampicillin-cloxacillin* ^NR^	1 (10)	2 (7)		3 (5)	

^1^ Not recommended for use in animals; ^A^ Access category antibiotic on WHO AwaRe list antibiotics; ^W^ Watch category antibiotic on WHO AwaRe list antibiotics; ^NR^ Not recommended on WHO AwaRe list of antibiotics; AM—Antimicrobials; WHO—World Health Organisation; CIA—Critically Important Antimicrobials; HIA—Highly Important Antimicrobials.

**Table 5:** Typology and description of the medicine-providers in the different countries whose interviews generated data for the analyses presented in this study.

Country	Type of Medicine-Provider	Definition
Uganda	Human or veterinary drug shops	Recognised drug outlets in the private for-profit sector, registered and licensed by the National Drug Authority to sell class “C” medicines (a restricted list of medicines including some antibiotic formulations) [38]. These drug outlets are licensed to provide either human or veterinary medicines exclusively.
Tanzania	Human or veterinary drug shops	The Tanzanian system distinguishes between type 1 (working under the supervision of a registered pharmacist) or type 2 (supervised by any person who has attended a five weeks’ accredited drug dispensing outlet training course) drug providers [40]. Type 1 providers can sell prescription only (including antibiotics), pharmacy only and general sale list (GSL) drugs. Type 2 providers can dispense GSL drugs and some antibiotics with prescription. Both type 1 and type 2 sell exclusively human or veterinary drugs.
	Community health worker	Community residents who have a close understanding of key aspects of the community (e.g., language, socio-economic status, and life and health experiences) [63]. They receive pre-service training to perform activities related to health promotion and disease prevention in the community. They cannot administer medicines but can refer patients to health facilities and accredited drug dispensing outlets to receive treatment, including antibiotics [64].
	Nurse	Nurses include nursing officers, nurse midwives, public health nurses. Training requirements include four years of secondary education followed by three years of professional training [65].
India	Private veterinarian	A self-employed worker who has received a university degree in veterinary medicine
	Public-private veterinary paraprofessionals (Pranibandhu and Animal Development Volunteer)	Public capacity – provide artificial insemination and livestock development services, paid on commission Private capacity – delivered livestock healthcare informally (including provision of antibiotics) and are paid directly by livestock-keepers
	Para-vet	A self-employed animal health worker informally trained in primary veterinary care
	Veterinary drug shop	A shop that sells allopathic medicines that are manufactured with the intention of animal consumption
	Human drug shop	A shop that sells allopathic medicines that are manufactured with the intention of human consumption
	Informal provider of human health	A self-employed health worker who does not hold a medical degree but is informally trained in the practice of human medicine
	Homeopath	A self-employed health worker in homeopathic medicine

Definitions for Indian providers adapted from Arnold et al. 2021 [26].

**Table 6:** Number of transcripts included in this analysis per country, by interview method and interviewee type.

Type of Interviewee	Number of Transcripts Received and Interview Method				Number of Transcripts with Crossover-Use Mentioned and Interview Method			
	Total	Uganda	Tanzania	India	Total	Uganda	Tanzania	India
Medicine-provider	41	7 FGD	8 IDI	26 IDI	30	7 FGD	6 IDI	17 IDI
Livestock-keeper	53	-	16 FGD 2 IDI	34 IDI 1FGD	23	-	11 FGD 1 IDI	10 IDI 1 FGD
Key informant	6	-	-	6 IDI	2	-	-	2 IDI
Community health worker	8	-	8 IDI	-	4	-	4 IDI	-
**Total**	**108**	**7**	**34**	**67**	**59**	**7**	**22**	**30**

IDI—In depth interviews. FGD—Focus group discussions. Data from Uganda and Tanzania were screened for accounts of crossover-use before sharing, the data from India were not. Data collection in India focused specifically on crossover-use, data from Uganda and Tanzania did not.

## Data Availability

Not applicable.

## Supplementary Materials

The following supporting information can be downloaded at: https://www.mdpi.com/article/10.3390/antibiotics11101342/s1, Table S1: Conditions for which veterinary antibiotics were used in humans in Uganda and Tanzania; Table S2: Human non-antibiotics used in animals for which condition in Uganda; Table S3: Human non-antibiotics used in animals for which condition in Tanzania; Table S4: Human non-antibiotics used in animals for which condition in India; Table S5: Conditions for which human antibiotics were used in animals in Uganda; Table S6: Conditions for which human antibiotics were used in animals in Tanzania; Table S7: Conditions for which human antibiotics were used in animals in India; Table S8: Methods from the four projects that provided data for this study.
