# Supplementary material for: Crossover-Use of Human Antibiotics in Livestock in Agricultural Communities: A Qualitative Cross-Country Comparison between Uganda, Tanzania and India
Source: Antibiotics (Basel). 2022 Sep 30;11(10):1342. doi: 10.3390/antibiotics11101342 (PMC9598773; doi:10.3390/antibiotics11101342)
Supplement: Supplementary file 1 [file antibiotics-11-01342-s001.zip › antibiotics-1863719-supplementary.pdf]

Supplementary Data

Table S1: Conditions for which veterinary antibiotics were used in humans in Uganda and Tanzania

**Country**	**Antibiotic**	**Use**	**Number of transcripts**
**Uganda**	Penicillin and streptomycin (combitic/penstrep)	Wounds	4
		Syphilis	2
		Gonorrhoea	1
	Penicillin	Gonorrhoea	2
	Tylosin (antibiotic feed additive)	Cough	1
**Tanzania**	Oxytetracycline spray	Treating wounds	2
	Penicillin and streptomycin (Penstrep)	Treating wounds	2
		Throat	1
	Oxytetracycline	Sore throat and ear infection	1
		For eyes	1
		For lung disease (according to respondent, not used anymore)	1

Table S2: Human non-antibiotics used in animals for which condition in Uganda

Drug	Animal species	Use	Number of transcripts
Artemether / Lumefantrine (Coartem)	Chicken	Fever	1
	-	-	1
Anti-retrovirals	Pigs	To fatten	2
Dexamethasone	-	-	1
Dextrose	Chicken	‘sick’	1
Diclofenac	Chicken	Fever	1
Panadol/paracetamol	Chicken	Fever	2
	Cow	Fever	1
Peramine	Chicken	Diarrhoea	1
Quinine	-	Fever	1

Table S3: Human non-antibiotics used in animals for which condition in Tanzania

Drug	Animal species	To treat	Number of transcripts
Furosemide	Cow	Fever	1
Hyoscine	Rats	To kill	1
Panadol/paracetamol	Chicken	Cough	1
		Newcastle	1
	-	-	1
Vitamin B	Cow	General health	1
	-		1

Table S4: Human non-antibiotics used in animals for which condition in India

Drug	Animal species	Use	Number of transcripts
Benadryl	Goat	Fever	1
		Bronchitis	1
Chromostat	Cow	Heavy menstrual bleeding/ retention of placenta	1
Diclofenac	Cow	Fever	1
	Goat	Fever	1
Digene syrup	-	-	1
Human allergic tablets	Cow	Swelling	1
Human digestive syrup	Goat	-	1
Meloxicam	-	-	1
Neopeptine	Dogs and cats	-	1
Omeprazole	-	-	1
Paracetamol	Goat	Fever	1
	-	-	2
Pheniramine (Avil)	-	-	1

Table S5: Conditions for which human antibiotics were used in animals in Uganda

Antibiotic	Animal species	Used for	Number of transcripts
Amoxicillin	Chicken	Cough and flu	1
		-	1
Ampicillin / cloxacillin	-	-	1
Chloramphenicol	Chicken	Diarrhoea	1
		Fowl typhoid	2
		Cough	2
		Fever	2
		-	1
Ciprofloxacin	Chicken	Fever	1
Enrofloxacin	-	-	1
Penicillin	-	-	1
Tetracycline	Chicken	Diarrhoea	1
		Cough	3
		Fever	1
		-	1
Trimethoprim / sulfamethoxazole	Chicken	Diarrhoea	1
	-	-	1

Table S6: Conditions for which human antibiotics were used in animals in Tanzania

Antibiotic	Animal species	Used for	Number of transcripts
Amoxicillin	Chicken	Newcastle disease	4
		Fowl pox	1
		Cough	1
		Diarrhoea	1
		Wounds	1
		Flu and pox	1
	-	-	1
		Cough	2
Ampicillin	Chicken	Wounds	1
	-	-	1
Erythromycin	Goat	Diarrhoea	1
	Chicken	-	2
	-	-	1
Oxytetracycline	Chicken	Cough	1
		Wounds in eye	1
Penicillin	Chicken	Newcastle disease	1
Tetracycline	Goat	Mucus	1
		Lung disease	1
	Chicken	Diarrhoea	1
	-	Lung disease	1
		Foot and mouth disease	1

Table S7: Conditions for which human antibiotics were used in animals in India

Antibiotic	Animal species	Used for	Number of transcripts
Amikacin	-	-	1
Amoxicillin	Chicken	Diarrhoea	1
	Goat	-	1
	-	-	2
Amoxicillin / clavulanic acid	Cattle	Mastitis	1
Amoxicillin / cloxacillin	-	-	1
Ampicillin	Goat	-	1
	-	-	1
Ampicillin / cloxacillin	Goat	Diarrhoea	1
Azithromycin	Bird	-	1
	Dog	-	1
Cefalexin	Cow	Wounds	1
	Goat	Wounds	1
	-	-	2
Cefotaxime	Cat	-	1
	Dog	-	1

Ceftriaxone	-	-	2
Ciprofloxacin	Goat	Cough	1
Chloramphenicol	Chicken	Fowl cholera	1
Doxycycline	-	-	2
Enrofloxacin	-	-	1
Gentamicin	Cow	-	2
	Goat	-	1
	Dog	-	1
Metronidazole	Chicken	Diarrhoea	2
	Goat	Diarrhoea	2
	Cow	Diarrhoea	1
Norfloxacin	Goat	Gastro-intestinal tract infection	1
		Diarrhoea	1
	Sheep	Gastro-intestinal tract infection	1
Norfloxacin / tinidazole	Goat	Diarrhoea	1
	Chicken	Diarrhoea	1
	-	Diarrhoea	1
Ofloxacin	-	-	1
Ofloxacin / ornidazole	Chicken	Diarrhoea	2
	Goat	Diarrhoea	1
Ornidazole	Goat	Diarrhoea	1
Oxytetracycline	Goat	-	1
	Cow	-	1
	-	-	1
Penicillin	-	Black quater	1
Sulfadimidine	-	-	2
Tetracycline	Chicken	Diarrhoea	1
	-	-	1
Trimethoprim / sulfamethoxazole	Chicken	Diarrhoea	1
	-	-	1

Table S8: Methods from the four projects that provided data for this study

Country	Study	Aim	Study site	Sampling	Data collection
**Uganda**	A comparison of antibiotic inventory, vendor knowledge, and perceptions of sector overlap between human and veterinary drug shops in Luwero, Uganda.	Develop a comparative knowledge of the antibiotic availability and relative popularity of the available products in human and veterinary drug shops, and contribute to a greater understanding of perceptions of sector similarity and overlap of use of these drugs in the community.	Luweero: rural agricultural community with a population of human and veterinary drug shops.	Documents obtained by the Luweero District Health Officer and District Veterinary Officer indicated 133 registered human and veterinary drug shops in the region. All identified veterinary drug shops in Luweero were approached, and these were matched geographically with 2 human drug shops. In total 66 drug shops were approached: 45 human and 21 vet. Vendors from 29 of these drug shops attended FGDs, forming 6 FGDs: 4 human DSV, and 2 vet DSV.	Data were collected in June and July 2018. The FGDs followed a semi-structured interview guide and the topics covered included: the DSVs role, common ABU in the community, perceptions of human and animal sector overlap, and the various challenges the DSVs encountered. The interview schedule was sufficiently flexible to allow for the emergence of unexpected topics as the discussions progressed. FGDs were conducted in Lugandan, with some English throughout. They were recorded and transcribed afterwards.
	Ensuring access to healthcare and medicines during COVID-19: critical challenges and feasible policy options for the medicines retail sector.	To understand and mitigate the vulnerability of supply and access to medicines within the Uganda health system.	Luweero and Mukono districts.	Sampling was done systematically from a list generated during mapping exercises and from the NDA. FGDs in Luweero included 6 Drug Shop Vendors; 2 with Pharmacy staff; 2 with clinic staff; 6 with community members; 1 with herbalists. FGDs in Mukono included; 3 with Drug Shop Vendors; 1 with Pharmacy staff; 4 with Clinic	FGDs in Luweero were done from 27/04/2021 to 02/06/2021. FGDs in Mukono were done between 18/08/2021 to 25/10/2021. Interview guides were developed covering the following topics: the impact of COVID-19 and the lockdown on a) practices in their shops/clinics/pharmacies and during any visits to the community b) current financial viability c) the viability of other

				staff; 6 with the community; 2 with herbalists The Medicine outlet survey was done in clinics, pharmacies and drug shops who had been open at least 3 months, with persons who sold medicines at the outlet at least half the open hours.<br>Number of medicine outlets – 624. Outlets per district: Luweero – 263 outlets Mukono – 362 outlets Types of medicine outlet: Drug shops - 53%; Pharmacies - 11% ; Clinics – 36%.	shops in the area d) access to medicines (particularly antibiotics, malaria medicine and contraceptives) e) mitigation strategies put in place to enable ongoing trading (protective measures, changes in business strategies) f) forms of collective action among MRS members to protect themselves, their businesses and others g) ways in which they would like to be part of the national response to COVID-19.
**India**	"If It Works in People, Why Not Animals?": A Qualitative Investigation of Antibiotic Use in Smallholder Livestock Settings in Rural West Bengal, India Part of 'A multi-stakeholder approach towards operationalising antibiotic stewardship in India's pluralistic	Investigate the provision of animal healthcare and ABU in smallholder livestock systems, the drivers of ABU, and the crossover-use of veterinary and human antibiotic formulations in humans and livestock in two village clusters of rural West Bengal, India.	West Bengal chosen as 85% of households owned livestock. Six rural villages in south parganas, West Bengal were purposively chosen because they had more than 50% of households with livestock, and few veterinary professionals (1 per 85,915 people)	KI, LK and AP were purposively sampled for their insights into antibiotic usage.	Semi-structured interview guides were developed for KIs, APs, and LKs. Adjustments were made throughout the data collection process to capture richer detail.<br>In-depth interviews were conducted by three researchers trained in qualitative methods, accompanied by a local research assistant. Interviews were conducted in Bangla and were recorded when consent was received.<br>Data were collected during June 2019 – January 2020.

	rural health system.				
**Tanzania**	Supporting the National Action Plan for Antimicrobial Resistance (SNAP-AMR) in Tanzania.	To provide novel insights into biological, social and cultural drivers of AMR within and out of hospitals at individual (inherent), micro- (community), meso- (institutional) and macro- (policy) level to prioritise use of limited human and financial resources in targeting evidence-based levers of behavioural change that will reduce the risk and burden of AMR.	Three districts (Mwanga, Ngorongoro and Misungwi) in three regions (Kilimanjaro, Arusha and Mwanza) of northern Tanzania representative of key livelihood strategies predominant in rural East Africa (rural smallholder, pastoral and agro-pastoral, respectively)	Two villages were selected in each district matched based on human/livestock population sizes, number of sub-villages and access to healthcare in the form of hospitals/dispensaries, veterinary officers and drug shops.<br>Study participants were selected through convenience sampling with sample sizes based on number of facilities and providers, and overall willingness to participate.	Two topics, animal or human health, were addressed in focus group discussions (FGDs) and in-depth interviews (IDIs), each discussed with either the respective healthcare providers or members of the community.<br>All interviews were conducted or moderated by Tanzanian research assistants fluent in the main languages spoken in the study locations, Swahili or Maasai. Interview protocols were developed to steer the discussion but retained a degree of flexibility in order to capture the natural flow of the conversation. Notes were taken throughout the FGDs/IDIs by dedicated note takers. The conversations were also audio recorded with subsequent transcription in Swahili and translation in English.

FGD - Focus group discussion
DSV – Drug shop vendor
ABU – Antibiotic use
KI – Key informants
LK – Livestock keepers
AP – Antibiotic providers
MRS – Medicines Retail Sector
